# Photocatalytic Activity of S-Scheme Heterostructure for Hydrogen Production and Organic Pollutant Removal: A Mini-Review

**DOI:** 10.3390/nano11040871

**Published:** 2021-03-30

**Authors:** Alexandru Enesca, Luminita Andronic

**Affiliations:** Product Design, Mechatronics and Environmental Department, Transilvania University of Brasov, Eroilor 29 Street, 35000 Brasov, Romania; andronic-luminita@unitbv.ro

**Keywords:** semiconductors, S-scheme heterojunction, hydrogen production, organic pollutant, photocatalysis

## Abstract

Finding new technologies and materials that provide real alternatives to the environmental and energy-related issues represents a key point on the future sustainability of the industrial activities and society development. The water contamination represents an important problem considering that the quantity and complexity of organic pollutant (such as dyes, pesticides, pharmaceutical active compounds, etc.) molecules can not be efficiently addressed by the traditional wastewater treatments. The use of fossil fuels presents two major disadvantages: (1) environmental pollution and (2) limited stock, which inevitably causes the energy shortage in various countries. A possible answer to the above issues is represented by the photocatalytic technology based on S-scheme heterostructures characterized by the use of light energy in order to degrade organic pollutants or to split the water molecule into its components. The present mini-review aims to outline the most recent achievements in the production and optimization of S-scheme heterostructures for photocatalytic applications. The paper focuses on the influence of heterostructure components and photocatalytic parameters (photocatalyst dosage, light spectra and intensity, irradiation time) on the pollutant removal efficiency and hydrogen evolution rate. Additionally, based on the systematic evaluation of the reported results, several perspectives regarding the future of S-scheme heterostructures were included.

## 1. Introduction

In recent years, due to the expansion of industrial activities, the problem of energy demands and water contamination have become major issues to be solved by the scientific community in order to increase the quality of life and human health [1,2]. The water contamination represents an important problem considering that the quantity and complexity of organic pollutant (such as dyes, pesticides, pharmaceutical active compounds, etc.) molecules can not be efficiently addressed by the traditional wastewater treatments [3,4]. The use of fossil fuels has disadvantages such as environmental pollution and limited stock, which inevitably causes energy shortages in various countries [5,6]. Replacing fossil fuel with hydrogen may be the key to energy sustainability if feasible production technologies and storage methods will be implemented.

A possible answer to the above issues is represented by the photocatalytic technology, which is characterized by the use of light energy in order to degrade organic pollutants or to split the water molecule into its components. Fast charge carriers recombination and limited light absorption spectra represent key issues of the mono-component photocatalysts [7,8]. The development of heterostructure photocatalysts has the advantage of using an extended light spectra, lower recombination rate, and higher charge carrier concentration. The heterostructure components are represented by semiconductors with various composition and morphology. Their ability to combine between them based on the energetic levels will determine the junction mechanism and photocatalytic efficiency [9,10,11]. 

**Figure 1:**
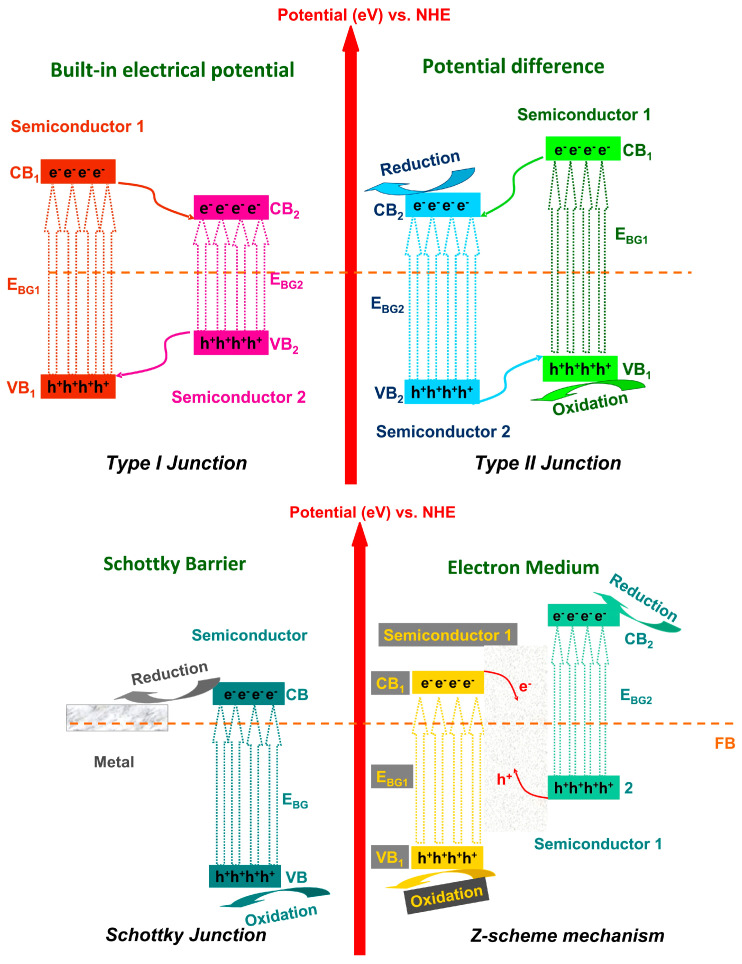
Energy diagrams of type I junction, type II junction, Schottky junction, and Z-scheme mechanism.

The literature mentions four typical mechanisms to describe heterostructures: type I, type II, Schottky junctions, and Z-scheme heterostructures (see Figure 1). However, in the recent period, the S-scheme heterostructure has emerged as a suitable candidate for highly efficient photocatalytic applications. Shortly, the type I junction works similar as a photovoltaic cell mechanism where the charge carriers are involved in redox reactions [12,13]. The type II junction uses the potential difference between the components to separate the photogenerated charges, preventing subsequent recombination [14,15]. The Schottky junctions benefits from the semiconductor–metal interface that is able to induce an effective mechanism to reduce charge carriers recombination and to increase the spectral light absorption [16,17]. The Z-scheme is represented by two photocatalytic systems connected together in order to enrich the charge carriers with stronger reduction/oxidation potential [18,19]. The S-scheme will be further explained in detail, including analogies with the traditional mechanisms.

The present mini-review aims to outline the most recent achievements (2019–2021) in the production and optimization of S-scheme heterostructures for photocatalytic applications. There may be many others scientific papers reporting similar or different information that are not included here due to the space limitation. The paper focuses on the influence of heterostructure components and photocatalytic parameters (photocatalyst dosage, light spectra and intensity, irradiation time) on the pollutant removal efficiency and hydrogen evolution rate. Additionally, based on the systematic evaluation of the reported results, several perspectives regarding the future of S-scheme heterojunctions were included.

## 2. S-Scheme Heterojunction Mechanism 

The need to adopt sustainable energy sources and remedy wastewater environmental issues has encouraged the researchers to find new materials and technologies that are able to give alternatives to the traditional ones. Semiconductors are considered as versatile materials that have or can be optimized to exhibit photocatalytic properties, alone or in combination with other materials [20,21,22]. The photocatalytic activity toward organic pollutant removal represents an advanced oxidation process, which is based on the efficient use of light energy to promote species that are able to induce partial or total decomposition. Heterostructures composed on various semiconductors such as oxides, sulfides, or organic have been investigated and optimized for specific applications [23,24,25].

**Figure 2:**
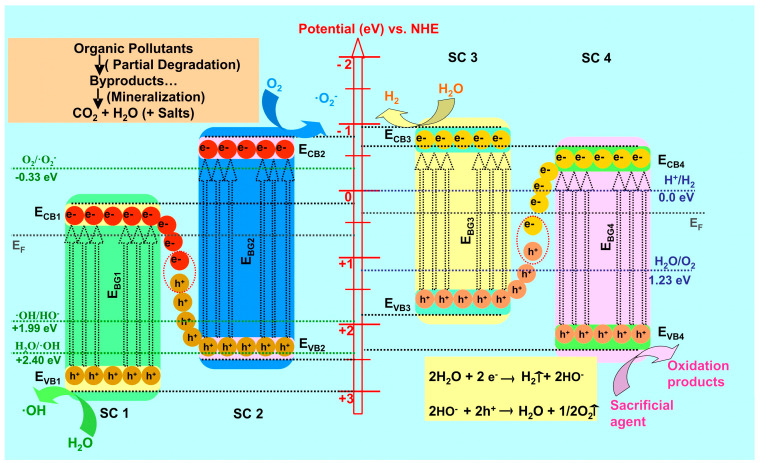
The S-scheme heterostructure mechanism for photocatalytic organic pollutant removal and hydrogen production.

Recently, the S-scheme heterojunctions mechanism (Figure 2) containing two n-type semiconductors materials was described by Yu et al. [26,27]. This structure is able to induce the partition (or a certain flux vector) and migration of the photogenerated electrons and holes, preserving at the same time their redox ability under the influence of an internal electric field due to the differences of the Fermi level corresponding to each semiconductor partner [28,29].

The S-scheme heterostructure has emerged as a suitable solution for the limitations induced by the previous photoactive mechanisms that use two or more semiconductors, coupled together using hydrothermal [30], solvothermal [31], sol–gel [32], precipitation [33], reduction [34], or other methods. Among these heterojunctions, a type-I heterostructured photocatalyst is not suitable to efficiently reduce the photogenerated charge carriers recombination because of an embedded band alignment established between the semiconductors components [35,36,37]. 

Figure 2 presents the S-scheme mechanism for organic pollutant removal and hydrogen production. The photogenerated electrons from the semiconductors 1 and 4 (SC1 and SC4) characterized by lower conduction band (CB) potential will combine (red dotted circles) with the photogenerated holes corresponding to semiconductor 2 and 3 (SC2 and SC3), having higher valence band (VB) potential and being eliminated at the interface. These charges do not have the ability to induce the formation of oxidative species or to participate directly in the organic molecule mineralization. The photogenerated holes in the higher valence band potential of one material and the photogenerated electrons in the higher conduction band potential of the other material remain. The process uses the driving force of the internal electric field, promoting the spatial separation of charge carriers under irradiation and preserving the strong redox ability [38,39,40]. 

If compared with the Z-scheme heterostructure, the S-scheme mechanism is driven by the built-in electric field that is able to reduce the transmission distance of photogenerated carriers based on the synergetic interface between the two semiconductors [41,42]. The Z-scheme disadvantage is represented by the longer transmittance distance that occurs with charge losses during the migration process. The S-scheme heterostructure advantage is given by the efficient use of the charge carriers due to the built-in electric field, which is created by the charge density difference between the semiconductors forming the junction. The built-in electric field can benefit from the elimination of comparatively useless photogenerated holes in the valence band and electrons in the conduction band, which can not participate in the formation of oxidative species due to the lower potential of the originating band energy. The remaining useful charge carriers have high redox ability (•O_2_^−^ from reduction reactions and •OH from oxidation reactions) for photochemical reactions [43,44].

The type-II heterostructure weakens the redox capabilities of the photogenerated electrons and holes with detrimental effect on the hydrogen production applications. Contrary, the S-scheme mechanism can maintain the photogenerated carriers with firm redox capability distinctly on two semiconductors, which is beneficial for hydrogen evolution during the water splitting process [45,46,47]. 

## 3. S-Scheme Heterostructure Photocatalytic Applications

This mini-review has selected two major photocatalytic applications of the S-scheme heterostructures: organic pollutant removal from wastewater and hydrogen production. It should be mentioned that there may be other papers that are not included here due to the space limitation or insufficient experimental data required to describe or compare the results. There are also papers describing the use of S-scheme heterostructures in other applications that were not considered in this work.

Photocatalytic technology is based on a chemical reaction mediated by mono-component or composite semiconductors that efficiently use the light radiation to promote the reduction and oxidation (redox) reaction rates due to the photogenerated carriers [48,49,50]. If the conduction band potential is between +0.5 and −2.0 V versus the normal hydrogen electrode (NHE), the photogenerated electrons will act as reduction agents based on their oxidizing capabilities [51,52]. The photocatalytic technology includes several steps: (i) photogeneration of charge carriers during the light irradiation, (ii) the diffusion of photogenerated carriers on the photocatalysts surface/interface, and (iii) the redox reaction on the photocatalysts surface [53,54,55].

### 3.1. Photocatalytic Removal of Organic Pollutant

**Figure 3:**
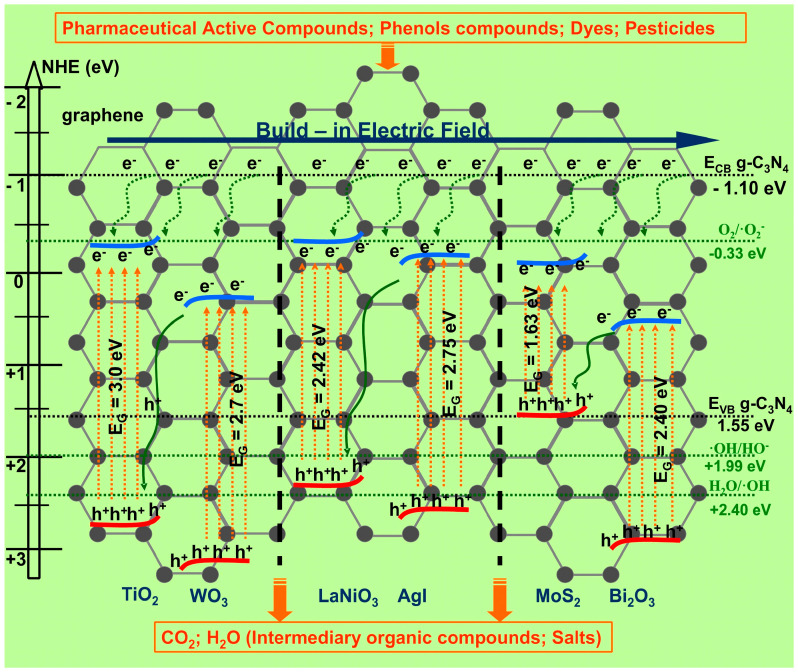
Energy band diagrams of TiO_2_/WO_3_, LaNiO_3_/AgI, and MoS_2_/Bi_2_O_3_ S-scheme heterostructures including the graphene structure (gray dots) and g-C_3_N_4_ band energy potential (black dots lines).

As presented in Figure 3, the S-scheme heterostructures induce the photocatalytic decomposition of organic compounds due to the photogenerated carriers that are trapped by dissolved oxygen and water molecules [56,57]. During irradiation, hydroxyl (•OH) and superoxide (•O_2_^−^) radicals are developed. These highly reactive oxygen species will decompose the organic pollutants to complete mineralization (if they contain only carbon, oxygen, and hydrogen atoms). Most of the organic pollutants contain also other atoms, and the formation of additional by-products is possible [58,59]. Table 1 presents the most recent studies regarding the S-scheme heterostructure application for organic pollutant removal.

#### 3.1.1. Heterostructures Obtained by Solvothermal Method

**Figure 4:**
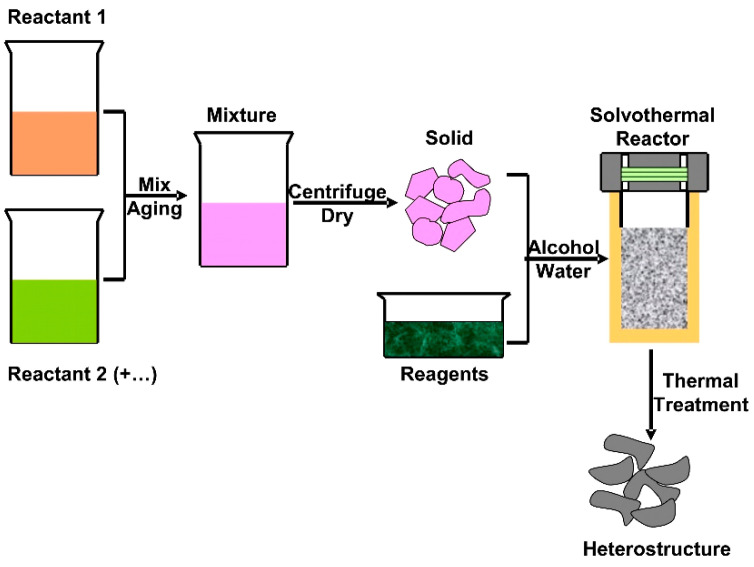
The solvothermal method for heterostructure development.

Pharmaceutical active compounds are helpful tools to eradicate human diseases but the pollution induced by the metabolized or partially metabolized pharmaceutical wastes represents an important bio-hazard [60,61]. The solvothermal technique (Figure 4) was used to develop three S-scheme heterostructures for tetracycline (TC) removal: black phosphorus (BP)/BiOBr [62], SnFe_2_O_4_/ZnFe_2_O_4_ [63], and TiO2/W_18_O_49_ [64]. The highest photocatalytic efficiency (93.2%) was recorded for SnFe_2_O_4_ nanoparticles (E_G_ = 1.88 eV)/ZnFe_2_O_4_ nanoparticles (E_G_ = 1.78 eV) heterostructure with a specific active surface of 68.79 m^2^/g. The SnFe_2_O_4_/ZnFe_2_O_4_ shows enhanced Vis light absorbance and a direct S-scheme path of charge separation and transfer. However, the TC concentration was 5× lower (10 mg/L) compared with BP nanosheets (E_G_ = 1.68 eV)/BiOBr nanosheets (E_G_ = 2.73 eV) photocatalytic activity, where the efficiency was 85%. The two-dimensional BP/BiOBr nano-heterojunction has a good performance on boosting the spatial charge separation in order to use holes–electrons with higher redox ability. The TiO_2_ nanosheets (E_G_ = 3.00 eV)/W_18_O_49_ spindle-like (E_G_ = 2.78 eV) heterostructure was able to remove 80.3% from the initial TC concentration (30 mg/L). In order to have an objective evaluation of the photocatalytic activity, the pollutant concentration, photocatalyst dosage, and irradiation time must be correlated. Based on these characteristics, the BP/BiOBr using a 100 mg/100 mL photocatalytst dosage was able to remove the highest tetracycline (TC) quantity (42.5 mg/L) during 90 min of irradiation with 300 W Vis light compared with SnFe_2_O_4_/ZnFe_2_O_4_ (9.3 mg/L in 120 min) and TiO_2_/W_18_O_49_ (22.5 mg/L in 75 min). The TiO_2_/W_18_O_49_ heterostructure was comparatively evaluated for Rhodamine B (RhB) photocatalytic removal, and the efficiency was higher (82.1%) than that of TC removal.

Similar studies for RhB photocatalytic removal were done with NiO/BiOI [65] and BiVO_4_@MoS_2_ [66] S-scheme heterostructures obtained by the solvothermal method. After 60 min of 300W Vis light irradiation, the NiO foam-like (E_G_ = 3.23 eV)/BiOI flower-like microspheres (E_G_ = 1.74 eV) exhibited 90% RhB photocatalytic removal (4.8 mg/L initial concentration). The photocatalytic efficiency remains unchanged after 5 cycles of 1 h each. The macroporous/microspheric hierarchical system allows light multi-scattering, which increases the photogenerated carrier’s concentration. The same photocatalytic efficiency (90%) was recorded for the BiVO_4_ nanorods @MoS_2_ sheets heterostructure but after 20 min of irradiation with 500 W Vis light intensity and using 20 mg/L RhB concentration. The BiVO_4_@MoS_2_ presents an improved photocurrent density that promotes the spatial distribution of BiVO_4_ oxidation sites and MoS_2_ reduction sites. The solvothermal method was also used to obtain Bi_2_O_3_ microspheres (E_G_ = 2.40 eV)/Bi_2_SiO_5_ flower-like (E_G_ = 3.64 eV) S-scheme heterostructure [67] with a specific active of 66.8 m^2^/g. The photocatalytic activity was evaluated against phenol and methyl orange (MO) organic pollutants at the same concentration (10 mg/L) and irradiation scenario (500 W Vis light). The results indicate an accelerated photocatalytic activity toward MO molecule (0.0026 min^−^^1^ rate constant, 67% efficiency) and lower oxidation rate on phenol (0.0001 min^−1^ and 30% efficiency). The heterostructure exhibit a small decrease of the phohotocatalytic efficiency after 5 cycles of 6 h each. The synergic effect induced by the heterostructure components enhances the production of predominant active radicals responsible for pollutant mineralization.

#### 3.1.2. Heterostructures Obtained by Hydrothermal Method

**Figure 5:**
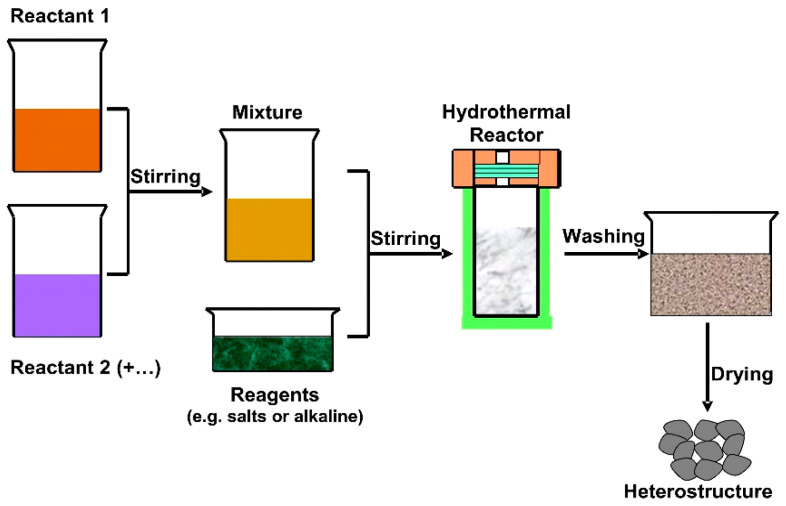
The hydrothermal method for heterostructure development.

The hydrothemal method (Figure 5) was employed to develop an S-scheme heterostructure for TC [68], 4-nitroaniline [69], and methylene blue (MB) [70] photocatalytic removal. After 50 min of irradiation with 300W Vis light, the WO_3_ nanorods (E_G_ = 2.76 eV)/CdIn_2_S_4_ nanosheets (E_G_ = 1.94 eV) were able to remove 95% of the initial TC concentration (50 mg/L). Compared with WO_3_, the CdIn_2_S_4_ shows strong Vis light absorption, suggesting that it is a good candidate to develop large-scale photocatalytic technologies. After 20 min of irradiation with a 500 W Vis light source, the CdS (E_G_ = 2.42 eV)/UiO-66 (E_G_ = 2.75 eV) removal efficiency reaches 80% toward the 4-nitroaniline molecule (20 mg/L). Using the pore-size performance of metal organic frameworks, the CdS/UiO-66 photocatalyst has a fast light excitation response and short carrier transport distances. The SnNb_2_O_6_ flaky structure (E_G_ = 2.10 eV)/Ag_3_VO_4_ nanoparticles (E_G_ = 2.16 eV) heterostructure behaves as an energy-efficient material that is able to remove 99% of MB (20 mg/L) in 10 min using a 50 W Vis light source (light emitting diode (LED)). The development of Ag_3_VO_4_ nanoparticles on 2D SnNb_2_O_6_ nanosheets favors the migration and separation of photogenerated carriers and increases the exposure of more active sites.

Photoreduction and hydrothermal coupled methods were involved in the development of a Bi_2_MoO_6_ (E_G_ = 2.64 eV)/g-C_3_N_4_ (E_G_ = 2.76 eV) S-scheme heterostructure [71] for RhB removal. Using a low photocatalyst dosage (5 mg/100 mL) and RhB concentration (5 mg/L), it was possible to remove 97.6% of the organic pollutant in 40 min under 300 W Vis light irradiation. The insertion of gold nanoparticles could act as a co-catalyst to promote the charge carriers mobility and facilitate the separation from the conduction band of g-C_3_N_4_. As mentioned before, the use of noble metals raises economic issues when up-scaling will be considered. The BiOI flakes (E_G_ = 1.68 eV)/Bi_2_WO_6_ nanosheets (E_G_ = 2.60 eV) S-scheme heterostructure [72] posseses the advantage of efficient Vis light absorption and facilitates the separation of photogenerated carriers based on the interfacial electric field between the flakes/nanosheets interface. The photocatalytic activity was higher toward RhB (90%, 0.0295 min^−1^) compared with MO (72%, 0.0027 min^−1^). The mechanism of pollutant degradation preference was not completely elucidated.

#### 3.1.3. Heterostructures Obtained by One-Pot and Precipitation Methods

An interesting study was done on an AgI nanoparticles (E_G_ = 2.75 eV)/I-BiOAc nanosheets (E_G_ = 2.35 eV) S-scheme heterostructure [73] obtained by a one-pot milling procedure. Using the same experimental conditions (500 W Vis light intensity, 20 mg/50 mL photocatalyst dosage), the degradation of four organic pollutants was evaluated. In the first step, methyl violet (MV) and bisphenol A (BPA) with 10 mg/L concentration were submitted to irradiation in the presence of AgI/I-BiOAc for 300 min. As expected, BPA reached lower degradation efficiency (71%) due to the molecule reluctance against oxidative species generated during the photocatalytic process. In contrast, the MV removal was up to 94.4%, which indicated a better surface affinity with the dye molecule. The stability evaluation indicates that the AgI/I-BiOAc photocatalytic properties decrease by 20% after 4 cycles. The second step validates the initial conclusion by showing that for other two dye molecules (MO and malachite green (MG)) with higher concentration (20 mg/L), the photocatalytic removal efficiency was 83% (MO) and 95% (MG). The typical S-scheme mechanism was favored by the formation of I-BiOAc solid solutions with suitable iodine content able to optimize the energy band structure, which converts a type-I AgI/BiOAc heterojunction into an S mechanism. A similar study involving three dye molecules (MG, RhB, and MO) and one antibiotic (TC) was done with a BiOBr nanosheets (E_G_ = 2.62 eV)/BiO(HCOO)Br-x tube (E_G_ = 2.96 eV) S-scheme heterostructure [74] obtained by the precipitation method. The two heterostructures use the same irradiation scenario, photocatalyst dosage, and pollutant concentration (20 mg/L). Additionally, both heterostructures have almost the same surface active area (9.0 m^2^/g for AgI/I-BiOAc and 9.65 m^2^/g for BiOBr/BiO(HCOO)Br-x). In terms of photocatalytic activity, the BiOBr/BiO(HCOO)Br-x reaches 100% MG degradation efficiency in 60 min. However, if MO is involved as a pollutant molecule, the photocatalytic efficiency decreases at 30% after 120 min of irradiation. These results demonstrate that the photocatalytic efficiency of the S-scheme heterostructure is dependent of the surface/interface chemistry with the pollutant molecule. The construction of interfacial-close-contact BiOBr/BiORBr allows the photo-generated electrons on the CB of BiOBr to migrate on the VB of BiORBr. Using charge carriers with higher redox capacity, the heterostructure produce oxidative species is able to induce 98% RhB and 80% TC photodegradation efficiencies.

The one-pot method was used to develop two g-C_3_N_4_ based S-scheme heterostructures for MO and MB photocatalytic removal. A Bi_2_S_3_ (E_G_ = 1.3 eV)/MoO_3_ (E_G_ = 3.01 eV)/g-C_3_N_4_ (E_G_ = 2.63 eV) lump-like heterostructure [75] has the advantage of double S-scheme heterojunctions and an active surface area of 43.2 m^2^/g. The cumulative photogenerated electrons present in the conduction band of C_3_N_4_ and Bi_2_S_3_, and the photogenerated holes will enrich in the valence band of MoO_3_. Additionally, the cumulative photoinduced holes with positive potentials (3.4 eV) in the valence band of MoO_3_ can be directly involved in the oxidation reaction of MO. Consequently, after 120 min of light irradiation (500 W, Vis), the photocatalytic removal efficiency of MO (10 mg/L) in the presence of Bi_2_S_3_/MoO_3_/g-C_3_N_4_ (50 mg/50 mL photocatalyst dosage) was 78% (0.0091 min^−1^). The Bi_2_S_3_ nanorods (E_G_ = 2.50 eV)/porous g-C_3_N_4_ nanosheets (E_G_ = 2.70 eV) heterostructure [76] was tested using a lower Vis light source intensity (50 W), shorter irradiation period (90 min), higher pollutant concentration (MB, 20 mg/L), and similar photocatalyst dosage. The results indicate that this heterostructure attempts 90% (0.0199 min^−1^) MB removal with lower energy consumption and a faster degradation rate, which significantly increases the technological sustainability. The photogenerated holes on the Bi_2_S_3_ valence band can directly mineralize the MB molecule. The potential of photoinduced electrons on the g-C_3_N_4_ conduction band is −1.21 eV, which is lower than the O_2_/•O_2_^−^ reduction potential (−0.33 eV). Consequently, the S-scheme has an important contribution in promoting the MB photocatalytic degradation.

#### 3.1.4. Heterostructures Obtained by Other Methods

The photocatalytic removal of ciprofloxacin and TC was evaluated using WO_3_ (E_G_ = 2.34 eV)/g-C_3_N_4_ (E_G_ = 2.65 eV) nanosheets [77] and BiOBr (E_G_ = 2.83 eV) nanosheets/BiOAc_1−x_Br_x_ (E_G_ = 3.28 eV) flower-like [78] S-scheme heterostructure. The WO_3_/g-C_3_N_4_ sample obtained by the template-assisted polymer method exhibited a 43.03 m^2^/g active surface area using the combined advantage of a g-C_3_N_4_ thin planar structure and WO_3_ oxygen deficit, which enhanced the charge transfer and increased the quantum efficiency. Based on these characteristics, the WO_3_/g-C_3_N_4_ photocatalyst in 50 mg/50 mL dosage is able to remove 90.54% of TC (20 mg/L) after 60 min of irradiation with a 300 W Vis light source. The stability tests indicate that the WO_3_/g-C_3_N_4_ photocatalytic efficiency remains unchanged after 6 cycles. The second heterostructure with a 33.9 m^2^/g active surface area obtained by co-precipitation benefits from the formation of BiOAc_1−x_Br_x_ solid solution, which enlarges the visible light response of BiOAc and prolongs the lifetime of photogenerated carriers with stronger redox ability. Using a 20 mg/50 mL photocatalyst dosage, the TC (20 mg/L) removal efficiency was 99.2% after 120 min of irradiation with a 500W Vis source. Comparing the rate constant of the two experiments, it became obvious that WO_3_/g-C_3_N_4_ possesses a faster removal rate (0.0378 min^−1^) than that of BiOBr/BiOAc_1−x_Br_x_ (0.0230 min^−1^). However, the photocatalytic activity of BiOBr/BiOAc1−xBrx increases when TC is replaced with RhB. In the same experimental conditions, the photocatalytic efficiency for RhB removal was 99.4%, and the rate constant was 0.0330 min^−1^.

Ciprofloxacin (CIP) is an extensively used antibiotic for a large number of bacterial infections. The LaNiO_3_ (E_G_ = 2.41 eV) nanoparticles/TiO_2_ (E_G_ = 3.20 eV) nanoparticles S-scheme heterostructure [79] obtained by the sol–gel method was employed to remove 10 mg/L CIP using a photocatalyst dosage of 50 mg/50 mL. Due to the spectral absorbance limitation of TiO_2_, the irradiation was done using both UV (300W Hg) and Vis (300 W Xe) sources during 210 min. The development of a binary structure can hinder the crystal grain growth and particles agglomeration, inducing the formation of a smaller particle size; it is also able to reduce the photogenerated carriers transfer route from the semiconductor inner to the surface and to produce enough oxidative species in order to reach 54% CIP photodegradation. The experiment was repeated using MO instead of CIP, and the results were even better. At 20 mg/L MO concentration, the photocatalytic efficiency after 150 min of irradiation was 92%. Using half of the MO concentration, the LaNiO_3_/TiO_2_ is able to completely remove the organic pollutant when a 100 mg/50 mL photocatalyst dosage is used. Similar results were obtained for RhB removal with g-C_3_N_4_ (E_G_ = 2.83 eV) nanosheets/Bi nanoparticles/BiVO_4_ (E_G_ = 2.40 eV) nanoparticles S-scheme heterostructure [80] with 50 m^2^/g specific active surface obtained by an in situ reduction process. After 70 min of irradiation with a 350W Vis light source, the g-C_3_N_4_/Bi/BiVO_4_ photocatalyst (50 mg/50 mL) can completely eliminate the RhB (10 mg/L) from the aqueous solution. It is worth noting that this heterostructure uses three active Vis light absorption centers (g-C_3_N_4_ nanosheets, BiVO_4_, and surface-decorated metallic Bi nanoparticles) inducing the direction of the built-in electric field from the g-C_3_N_4_ surface to the BiVO_4_ surface, which favors the vectorial interfacial transition of the photogenerated carriers from the BiVO_4_ conduction band to the g-C_3_N_4_ valence band during the photocatalytic activity.

A comparative study of the photocatalytic removal efficiency of three dye molecules (RhB, MO, and acid orange II (AO II)) was done on a g-C_3_N_4_ (E_G_ = 2.70 eV) nanosheets/ZrO_2_ (E_G_ = 2.60 eV) nanoparticles S-scheme heterostructure [81] obtained by the calcination method. The heterostructure has a large surface area (116.4 m^2^/g) due to the dispersed nanoparticles being able to provide more active sites and higher Vis light absorption capacity. The g-C_3_N_4_ structure has the ability to enlarge the distribution area. All these factors will be beneficial on the production of reactive free radicals during the 150 min of irradiation with a 300 W Vis light source. Using the same experimental conditions in terms of pollutant concentration (10 mg/L), photocatalyst dosage (30 mg/50 mL), and irradiation scenario, the photocatalytic removal efficiencies were 98% for AO II, 82% for RhB, and 50% for MO. The efficiency differences are mainly attributed to the dye molecule and surface chemical compatibility. The S-scheme charge transfer mechanism is based on the smaller work function (4.18 eV) and higher Fermi level of g-C_3_N_4_, contrary to the ZrO_2_, which will enhance the photocurrent response and decrease the emission PL spectrum intensity.

Two Bi_2_O_3_ based S-scheme heterostructures were developed to remove ethinylestradiol and phenol. The photodeposition method was used to obtain Bi_2_O_3_ (E_G_ = 2.80 eV) nanoplates/CuBi_2_O_4_ (E_G_ = 1.87 eV) nanoparticles/Ag nanoparticles heterostructure [82], and the photocatalytic activity was evaluated against 17-α ethinylestradiol (10 mg/L), which is an estrogen medication used in hormone-sensitive cancers. The S-scheme mechanism is based on ternary composite materials, which use the smooth passage of photogenerated electrons from the Bi_2_O_3_ conduction band to the CuBi_2_O_4_ valence band, while silver metal is an electron reservoir as well as surface plasmon, ensuring the achievement of higher charge carrier separation. Based on this mechanism, the photocatalyst (40 mg/100 mL) reaches 94.6% ethinylestradiol removal efficiency after 120 min of irradiation with a 250W Vis light source. A lower photocatalytic efficiency (50%) was obtained for TC removal using a Bi_2_O_3_ (E_G_ = 2.77 eV) rod-like/TiO_2_ (E_G_ = 3.0 eV) nanofiber S-scheme heterostructure [83]. The photocatalyst was obtained by in situ photoreductive deposition, and the surface are was 51 m^2^/g. The photocatalytic experimental conditions use extreme values in terms of pollutant concentration (100 mg/L) and photocatalyst dosage (50 mg/15 mL). As mentioned before describing other similar reports [67,73], the phenol compounds are more resistant to photocatalytic decomposition. The S-scheme mechanism uses photogenerated holes in TiO_2_ as recombination charges for the electrons from Bi_2_O_3_, resulting in more available photogenerated electrons in the TiO_2_ conduction band for •O_2_^−^ production. Another outcome is represented by the increased availability of holes on the Bi_2_O_3_ valence band, which favors the phenol oxidation by reactive oxidative species.

### 3.2. Photocatalytic Water Splitting for Hydrogen Production

**Figure 6:**
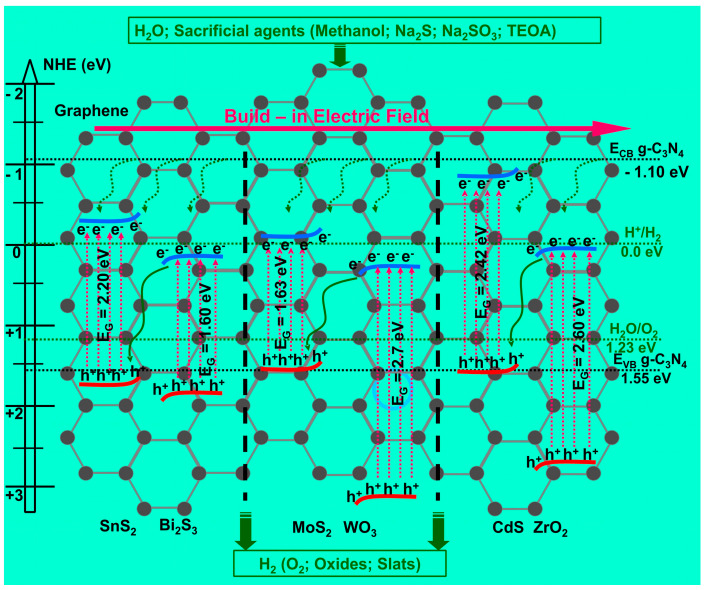
Energy band diagrams of SnS_2_/Bi_2_S_3_, MoS_2_/WO_3_, and CdS/ZrO_2_ S-scheme heterostructures including the graphene structure (gray dots) and g-C_3_N_4_ band energy potential (black dots and lines).

Photochemical (PC) and photoelectrochemical (PEC) water splitting are considered as potential candidates to generate clean and cost-effective hydrogen using a green pathway of solar energy conversion. However, there are still important difficulties to overcome: (i) limited light spectral absorption, (ii) efficient conversion of the photogenerated carriers during the water-splitting process, (iii) suitable conduction and valence band-edge potentials for redox reactions. Figure 6 presents the mechanism of water splitting during using S-scheme heterojunctions where the hydrogen potential is in the close proximity of the semiconductors conduction band. The potential required to generate water splitting (1.23 eV) can be obtained if the semiconductors partners have a suitable position of the energy bands. In order to increase the hydrogen production efficiency, sacrificial agents are used as favorite candidates for the oxidation process. Table 2 presents the most recent studies regarding the S-scheme heterojunction application in organic pollutant removal.

The hydrothermal method was employed to develop MoS_2_/CoAl [84] and WO_3_/TiO_2_/reduced graphene oxide (rGO) [85] heterostructures for hydrogen production using methanol aqueous solution. Both components of the MoS2/CoAl sample have the light absorption range in Vis spectra (E_GMoS2_ = 1.8 eV and E_GCoAl_ = 2.1 eV), and the heterostructure is able to exhibit 17.1 µmol/h hydrogen evolution under 300W light irradiation. Benefiting from the advantages of 2D/2D architecture and high photocurrent intensity, the MoS_2_/CoAl represents a promising candidate for hydrogen technology. The WO_3_/TiO_2_/rGO heterostructure have the disadvantage of using two semiconductors active in the UV range (E_GWO3_ = 3.2 eV and E_GTiO2_ = 2.6 eV). However, by inserting rGO in the heterostructure composition, it was possible to increase the specific surface area up to 165 m^2^/g and the absorption range. The hydrogen evolution rate (HyER) was optimized at 12.29 µmol/h using 50 mg/80 mL photocatayst dosage. The combined effect of the S-scheme heterostructure, formed between semiconductors metal oxides, and the Schottky heterojunction, formed between TiO_2_ and graphene sheets, is able to suppress the recombination of useful photogenerated carriers and increase the number of active surface sites for the reduction reaction.

An isotype g-C_3_N_4_ heterostructure consisting of hydrothermally treated melamine and urea was developed [86] to work as Vis photoactive catalysts. The melamine g-C_3_N_4_ (MCN) and urea g-C_3_N_4_ (UCN) with different morphologies (comb-like for MCN and laminar for UCN) but a compatible band structure (E_GMCN_ = 2.69 eV and E_GUCN_ = 2.81 eV) were irradiated with 300 W Vis light for 180 min. The photogenerated electrons can migrate from MCN to UCN due to the MCN large work function, resulting in a positive interface on MCN and negative interface on UCN. The stability test indicated that the HyER remains unchanged after 4 cycles. The HyER using a triethanolamine (TEOA) sacrificial agent was significant (29.9 µmol/h) considering the heterostructure specific surface area of 46 m^2^/g. Using the same sacrificial agent and synthesis method, the CoAl layered double hydroxides (LDH)@Ni-metal–organic frameworks (MOF)-74 [87] heterostructure exhibits 7× higher HyER (213 µmol/h) under lower irradiation intensity (5 W Vis LED). The CoAl LDH nanolayers loaded onto the surface of Ni-MOF-74 form an S-scheme heterostructure photocatalyst where electrons from CoAl LDH are transferred to Ni-MOF-74, accelerating the spatial separation and migration of the photogenerated charged with a positive impact of hydrogen evolution reaction.

Two S-scheme heterostructures with similar band gap values were obtained by hydrothermal [88] and chemical deposition [89] methods. The hydrothermal heterostructure is composed by SnNb_2_O_6_ nanosheets and CdS diethylenetriamine nanosheets with band gap values between 2.25 eV (SnNb_2_O_6_) and 2.51 eV (CdS). Using a photocatalyst dosage of 30 mg in 50 mL and Na_2_S + Na_2_SO_3_ as sacrificial agents, the heterostructure is able to produce 234.24 µmol/h HyER under 300 W Vis light irradiation. In similar experimental conditions in terms of light radiation and sacrificial agents, the Mn_0.5_Cd_0.5_S nanoparticles (E_G_ = 2.48 eV)/WO_3_ nanorods (E_G_ = 2.7 eV) heterostructure obtained by the chemical deposition method exhibits a significantly higher HyER (517.13 µmol/h) even if the photocatalyst dosage is lower (50 mg/100 mL). The reason consists of the Mn_0.5_Cd_0.5_S/WO_3_ multichannel-enhanced charge transfer combined with the Au nanoparticles insertion, which acts as an electron storage and provider for driving the hydrogen evolution reaction from water reduction. On the contrary, the SnNb_2_O_6_/CdS is a noble metal-free photocatalyst with interfaces toward a 2D S-scheme and with the potential to be cost effective for large-scale implementation.

One-pot [90] and thermal condensation [91] methods were employed to verify the photocatalytic properties of two S-scheme heterostructures in the presence of the TEOA sacrificial agent and 300 W Vis light intensity. The MoO_3_/g-C_3_N_4_ heterostructure obtained by the one-pot method is characterized by uniformly distributed MoO_3_ nanoparticles on the g-C_3_N_4_ nanosheets. The HyER reaches 25.62 µmol/h due to the g-C_3_N_4_ structure change taking place during the one-pot process, which will increase the Vis radiation absorption capacity and the charge carrier’s photogeneration. A higher HyER (32.92 µmol/h) corresponds to the S-doped g-C_3_N_4_ nanosheets/N-doped MoS_2_ nanobelts heterostructure obtained by thermal condensation and having a 4.9 m^2^/g specific surface area. It must be underlined that in this case, the photocatalyst dosage was double compared with the MoO_3_/g-C_3_N_4_ heterostructure, which may explain the HyER differences. Additionally, the N-doped MoS_2_ was involved not only as a co-catalyst during the H_2_ production but also to extend the light absorption spectra, facilitating the charge carrier transfer and separation through N-Mo and C-S-Mo chemical bonds.

The influence of photocatalyst dosage can be observed by comparing the HyER values of g-C_3_N_4_/CdS-diethylenetriamine [92] and Bi_2_S_3_/g-C_3_N_4_ [93] obtained by the solvothermal method and tested in similar conditions (Na_2_S + Na_2_SO_3_ sacrificial agents and 300 W Vis light radiation). The highest HyER value (486.9 µmol/h) corresponds to the g-C_3_N_4_ nanosheets (E_G_ = 2.61 eV)/CdS-diethylenetriamine nanosheets (E_G_ = 2.68 eV) heterostructure with a specific active surface of 36.6 m^2^/g. The synergic activity of the nanocomposite induces fast interfacial charge separation and diffusion of the photogenerated charge. The Bi_2_S_3_ nanorods (E_G_ = 1.60 eV)/g-C_3_N_4_ nanosheets (E_G_ = 2.78 eV) heterostructure attempts 101.8 µmol/h HyER, even if the specific active surface is higher (58.1 m^2^/g) due to the reduced photocatalyts dosage (30 mg/100 mL). However, the advantage of the second heterostructure is given by its composition characterized by earth-abundant elements with low environmental impact. Using the same sacrificial agents and irradiation conditions, the g-C_3_N_4_ nanosheets (E_G_ = 2.85 eV)/CdSe-amine flower-like (E_G_ = 1.86 eV) S-scheme heterostructure [94] obtained by the microwave-assisted solvothermal method reaches 18.8 µmol/h HyER. The g-C_3_N_4_/CdSe-amine sample is characterized by a 73.1 m^2^/g specific active surface, and the photocatalyst dosage was 20 mg/50 mL. The incorporation of Cl atoms will promote interlayer charge transfer and a conduction band up-shifted value. Additionally, the S atom can be used to replace the N atom into the CN framework, which allows tailoring the band gap.

Finally, the SnO_2_ nanoparticles (E_G_ = 3.7 eV)/SnS_2_ nanosheets (E_G_ = 2.2 eV) S-scheme heterostructure [95] was obtained by the solvothermal method, and the photocatalytic activity was evaluated in the absence of sacrificial agents. Under 300W Vis light irradiation, the HyER value was 5.5 µmol/h. The HyER remains unchanged after four cycles (3 h/cycle). The results indicate that it is possible to obtain a certain quantity of hydrogen without using other substances, which may have a detrimental environmental impact due to the oxidation products when the process targets large-scale applications. Using 0D SnO_2_ nanoparticles developed on the surfaces and edges of 2D SnS_2_ nanosheets will increase the carrier’s recombination rate corresponding to relatively weaker redox capacity and favor the separation of photoinduced carriers with stronger redox capacity.

These results indicate that the increase of HyER values can be obtained by correlating several simultaneous conditions: suitable position of the energy bands, specific active surface, catalyst dosage, and irradiation scenarios.

## 4. Conclusions and Perspectives

Finding new technologies and materials that provide real alternatives to the environmental and energy-related issues represents a key point in the future sustainability of the industrial activities and social development. Photocatalytic technologies possess several advantages: (i) environmentally “friendly” materials, (ii) renewable energy source, (iii) long life-time cycles, and (iv) high versatility for both wastewater treatment and hydrogen production. The use of environmentally friendly materials will be a significant contribution in implementing a sustainable new large-scale technology with good social acceptance and relatively easy integration in the wastewater plants.

The S-scheme heterostructure mechanism describes a new pathway to induce the partition and migration of the photoinduced charge carriers preserving their redox ability under the influence of an internal electric field due to the differences of the Fermi level corresponding to each semiconductor partner. Based on this pathway, several works were presented in order to evaluate the photocatalytic efficiency of the S-scheme heterostructure on organic pollutant removal and hydrogen production. The systemic evaluation indicates that the photocatalytic efficiency should be correlated with other aspects (pollutant type and concentration, photocatalyst dosage, irradiation scenario, etc.) in order to have a comprehensive view on the future feasibility of the process. The high photocatalytic efficiency with the expense of large energy or material consumption is not sustainable when future large-scale implementation is considered. Using low energy light sources (LED) allows eliminating 90% of MB (20 mg/L) using SnNb_2_O_6_/Ag_3_VO_4_ or Bi_2_S_3_/porous g-C_3_N_4_ S-scheme heterostructures. Similarly, a 5W light energy source can be used to irradiate the CoAl LDH@Ni-MOF-74 S-scheme heterostructure in order to reach a 213 µmol/h hydrogen evolution rate. Reducing the irradiation time and photocatalysis dosage is essential for increasing the overall efficiency of the photocatalytic process. The BiVO4@MoS2 heterostructure was able to remove 90% of RhB (20 mg/L) in 20 min. By increasing the irradiation time to 40 or 100 min in order to gain a few extra percentages may not be cost effective due to the higher energy consumption. The photocatalyst recovery as well as standardization procedures remain an important issue to be solved in the near future. 

The main advantages of using S-scheme heterostructures are related to the efficient transfer and conversion of photogenerated charge carriers in order to be involved in the mineralization reaction of organic pollutant. Additionally, the S-scheme is a useful tool to design future photocatalyt materials for the extensive use of a solar spectrum in various energy applications. The disadvantage is given by the limitations induced by the materials type that can be used and tailored to increase the conversion efficiency and light absorbance. 

As perspectives, the use of coupled techniques and procedures may increase the overall performance in both wastewater treatment and hydrogen production. There are already many papers showing the advantages of simultaneous adsorption–photocatalysis processes. Biodegradation may be considered as well based on the use on non-polluting materials and low energy costs.

The integration of new or optimized materials can contribute to the increase of the photocatalytic efficiency. Graphitic carbon nitride (g-C_3_N_4_) is a metal-free semiconductor and nontoxic photocatalyst exhibiting good chemical stability and suitable band gap, allowing the use Vis light energy. Other materials can be optimized by doping, surface sensitization, exfoliation, or defect engineering in order to improve their quantum efficiency and photocatalytic activity. Doping can target specific materials properties without affecting the entire structure and composition. It can be used to modify the conduction type, the absorbance spectra, or even the surface energy. The size of the photocatalytic materials plays an important role considering two aspects: area/volume ratio and photogenerated charge carriers migration. The photocatalytic process requires large surfaces on small volumes that favor the interface development of oxidative species.

## Figures and Tables

**Table 1:** Recent representative studies on the use of S-scheme heterojunction for organic pollutant removal.

Tandem Composition and Band Gap (E_G_)	Synthesis Method	Morphology and S_BET_ [m^2^/g]	Radiation Parameters		Photocatalytic Parameters					Ref.
			Light Spectra	Intensity [W]	Pollutant Concentration [mg/L]	Photocatalyst Dosage [mg/mL]	Time [min]	Efficiency [%]	Rate Constant [min^−1^]	
BP (black phosphorus) (1.68 eV)/BiOBr (2.73 eV)	Solvothermal	BP (black phosphorus) nanosheets/BiOBr nanosheets S_BET_ = np *	Vis	300 (Xe)	Tetracycline (TC) = 50 mg/L	100 mg/100 mL	90	85	0.021	[62]
SnFe_2_O_4_ (1.88 eV)/ ZnFe_2_O_4_ (1.78 eV)	Solvothermal	SnFe_2_O_4_ nanoparticles/ ZnFe_2_O_4_ nanoparticles S_BET_ = 68.76	Vis	np	TC = 10 mg/L	30 mg/100 mL	120	93.2	np	[63]
TiO_2_(3.0 eV)/W_18_O_49_ (2.78 eV)	Solvothermal	TiO_2_ nanosheets and W_18_O_49_ spindle-like S_BET_ = np	Vis	np	Rhodamine B (RhB) = 10 mg/L	np	75	82.1	0.0261	[64]
					TC = 30 mg/L			80.3	np	
NiO (3.23 eV)/BiOI (1.74 eV)	Solvothermal	NiO foam-like/BiOI flower-like microspheres S_BET_ = 20.7	Vis	300 (Xe)	RhB = 4.8 mg/L	0.04 mg/30 mL	60	90	0.057	[65]
BiVO_4_ (1.96 eV) @MoS_2_ (1.63 eV)	Solvothermal	BiVO_4_ nanorods @MoS_2_ sheets S_BET_ = np	Vis	500 (Xe)	RhB = 20 mg/L	np	20	90	0.111	[66]
Bi_2_O_3_ (2.40 eV)/Bi_2_SiO_5_ (3.64 eV)	Solvothermal	Bi_2_O_3_ microspheres/Bi_2_SiO_5_ flower-like microstructure S_BET_ = 66.8	Vis	500 (Xe)	Methyl orange (MO) = 10 mg/L	50 mg/50 mL	420	67	0.0026	[67]
					Phenol = 10 mg/L		360	30	0.0001	
WO_3_ (2.76 eV)/CdIn_2_S_4_ (1.94 eV)	Hydrothermal	WO_3_ nanorods/CdIn_2_S_4_ nanosheets S_BET_ = 32	Vis	300 (Xe)	TC = 50 mg/L	30 mg/30 mL	50	95	np	[68]
CdS (2.42 eV)/UiO-66 (2.75 eV)	Hydrothermal	CdS nanoparticles/UiO-66 nanoparticles S_BET_ = np	Vis	500 (Xe)	4-nitroaniline = 20 mg/L	40 mg/40 mL	20	80	0.085	[69]
SnNb_2_O6 (2.10 eV)/Ag_3_VO_4_ (2.16 eV)	Hydrothermal	SnNb_2_O_6_ flaky structure/Ag_3_VO_4_ nanoparticles S_BET_ = 58.24	Vis	50 (LED)	Methylene blue (MB) = 20 mg/L	30 mg/30 mL	10	99	0.2256	[70]
Bi_2_MoO_6_ (2.64 eV)/g-C_3_N_4_ (2.76 eV)	Photoreduction and hydrothermal	Bi_2_MoO_6_ sheet like/g-C_3_N_4_ nanosheets S_BET_ = np	Vis	300 (Xe)	RhB = 5 mg/L	5 mg/100 mL	40	97.6	0.0808	[71]
BiOI (1.68 eV)/Bi_2_WO_6_ (2.60 eV)	Hydrothermal	BiOI flakes/Bi_2_WO_6_ nanosheets S_BET_ = 23.31	Vis	500 (Xe)	RhB = 10 mg/L	50 mg/50 mL	150	90.1	0.0295	[72]
					MO = 10 mg/L			72.1	0.00217	
AgI (2.75 eV)/I-BiOAc (2.35 eV)	One-pot milling	AgI nanoparticles/I-BiOAc nanosheets S_BET_ = 9.0	Vis	500 (Xe)	Methyl violet (MV) = 10 mg/L	20 mg/50 mL	300	94.4	0.047	[73]
					Bisphenol A (BPA) = 10 mg/L	20 mg/50 mL	300	71.1	0.035	
					MO = 20 mg/L	20 mg/50 mL	120	83	np	
					Malachite green (MG) = 20 mg/L	20 mg/50 mL	60	95	np	
BiOBr (2.62 eV)/BiO (HCOO)Br-x (2.96 eV)	Precipitation	BiOBr nanosheets/BiO (HCOO)Br-x tube S_BET_ = 9.65	Vis	500 (Xe)	MG = 20 mg/L	20 mg/50 mL	60	100	0.064	[74]
					RhB = 20 mg/L		120	98	0.024	
					TC = 20 mg/L			80	np	
					MO = 20 mg/L			30		
Bi_2_S_3_ (1.3 eV)/MoO_3_ (3.01 eV)/g-C_3_N_4_ (2.63 eV)	One-pot solid-state reaction	Bi_2_S_3_/MoO_3_/C_3_N_4_ lump-like structure S_BET_ = 43.2	Vis	500 (Xe)	MO = 10 mg/L	50 mg/50 mL	120	78	0.0091	[75]
Bi_2_S_3_ (2.50 eV)/ porous g-C_3_N_4_ (2.7 eV)	One-pot	Bi_2_S_3_ nanorods/ porous g-C_3_N_4_ nanosheets S_BET_ = np	Vis	50 (LED)	MB = 20 mg/L	30 mg/30 mL	90	90	0.0199	[76]
WO_3_ (2.34 eV)/g-C_3_N_4_ (2.65 eV)	Template assisted polymer	WO_3_/g-C_3_N_4_ Nanosheets S_BET_ = 43.03	Vis	300 (Xe)	TC = 20 mg/L	50 mg/50 mL	60	90.54	0.0378	[77]
BiOBr (2.83 eV)/BiOAc_1−x_Br_x_ (3.28 eV)	Co-precipitation	BiOBr nanosheets/BiOAc_1−x_Br_x_ flower like S_BET_ = 33.9	Vis	500 (Xe)	TC = 20 mg/L	20 mg/50 mL	120	99.2	0.023	[78]
					RhB = 20 mg/L			99.4	0.033	
LaNiO_3_ (2.42 eV)/TiO_2_ (3.2 eV)	Sol-gel	LaNiO_3_ nanoparticles/TiO_2_ nanoparticles S_BET_ = np	UV-Vis	300 (Hg) 300 (Xe)	MO = 10–20 mg/L	100 mg/50 mL	150	100 (10 mg/L) 92 (20 mg/L)	np	[79]
					Ciprofloxacin (CIP) = 10 mg/L	50 mg/50 mL	210	54	np	
g-C_3_N_4_ (2.83 eV)/Bi/BiVO_4_ (2.4 eV)	In-situ reduction	g-C_3_N_4_ nanosheets/Bi nanoparticles/ BiVO_4_ nanoparticles S_BET_ = 50	Vis	350 (Xe)	RhB = 10 mg/L	50 mg/50 mL	70	100	0.067	[80]
g-C_3_N_4_ (2.7 eV)/ZrO_2_ (2.6 eV)	Calcination	g-C_3_N_4_ nanosheets/ZrO_2_ nanoparticles S_BET_ = 116.4	Vis	300 (Xe)	RhB = 10 mg/L	30 mg/50 mL	150	82	np	[81]
					MO = 10 mg/L			50		
					Acid orange II (AO II) = 10 mg/L			98		
Bi_2_O_3_ (2.8 eV)/CuBi_2_O_4_ (1.87 eV)/Ag	Photodeposition	Bi_2_O_3_ nanoplate/CuBi_2_O_4_ nanoparticles/Ag nanoparticles S_BET_ = np	Vis	250 (Xe)	17-α Ethinylestradiol = 10 mg/L	40 mg/100 mL	120	94.6	0.0185	[82]
Bi_2_O_3_ (2.77 eV)/TiO_2_ (3.0 eV)	In-situ photoreductive deposition	Bi_2_O_3_ rod-like/TiO_2_ nanofiber S_BET_ = 51	Vis	300 (Xe)	Phenol = 100 mg/L	50 mg/15 mL	120	50	np	[83]

* not provided.

**Table 2:** Recent representative studies on the use of S-scheme heterojunction for organic pollutant removal.

Tandem Composition and Band Gap (E_G_)	Synthesis Method	Morphology and S_BET_ [m^2^/g]	Radiation Parameters		Hydrogen Production				Ref.
			Light Spectra	Intensity [W]	Sacrificial Agent	Photocatalyst Dosage [mg/L]	Time [min]	Evolution Rate [µmol/h]	
MoS_2_ (1.8 eV)/CoAl (2.1 eV)	Hydrothermal	MoS_2_ spherical/CoAl carnations S_BET_ = np	Vis	300 (Xe)	np *	50 mg/80 mL	300	17.1	[84]
WO_3_ (3.2 eV)/TiO2 (2.6 eV)/rGO	Hydrothermal	WO_3_ nanoparticles/TiO_2_ nanoparticles/rGO nanosheets S_BET_ = 165	Vis	350 (Xe)	np	50 mg/80 mL	180	12.29	[85]
Melamine g-C_3_N_4_ (2.69 eV)/Urea g-C_3_N_4_ (2.81 eV)	Hydrothermal	Melamine g-C_3_N_4_ comb-like/Urea g-C_3_N_4_ laminar S_BET_ = 46	Vis	300 (Xe)	Triethanolamine (TEOA)	50 mg/100 mL	180	29.9	[86]
CoAl layered double hydroxides (LDH) (2.40 eV) @Ni- Metal–organic frameworks (MOF)-74 (2.37 eV)	Hydrothermal	CoAl LDH nanosheets @Ni-MOF-74 quadrilateral Structure S_BET_ = np	Vis	5 W (LED)	TEOA	10 mg/30 mL	350	213	[87]
SnNb_2_O_6_ (2.25 eV)/CdS diethylenetriamine (2.51 eV)	Hydrothermal	SnNb_2_O_6_ nanosheets/CdS diethylenetriamine nanosheets S_BET_ = 93.27	Vis	300 (Xe)	Na_2_S + Na_2_SO_3_	30 mg/50 mL	240	234.24	[88]
Mn_0.5_Cd_0.5_S (2.48 eV)/WO_3_ (2.7 eV)	Chemical deposition	Mn_0.5_Cd_0.5_S nanoparticles/WO_3_ nanorods S_BET_ = np	Vis	300 W (Xe)	Na_2_S + Na_2_SO_3_	50 mg/100 mL	180	517.13	[89]
MoO_3_/g-C_3_N_4_ (2.7 eV)	One-pot	MoO_3_ nanoparticles/g-C_3_N_4_ nanosheets S_BET_ = np	Vis	300 (Xe)	TEOA	50 mg/200 mL	480	25.62	[90]
S-doped g-C_3_N_4_ (2.80 eV)/N-doped MoS_2_ (1.80 eV)	Thermal polycondensation	S-doped g-C_3_N_4_ nanosheets/N-doped MoS_2_ nanobelts S_BET_ = 4.9	Vis	300 (Xe)	TEOA	50 mg/100 mL	240	32.92	[91]
g-C_3_N_4_ (2.61 eV)/CdS-diethylenetriamine (2.68 eV)	Solvothermal	g-C_3_N_4_ nanosheets/CdS-diethylenetriamine nanosheets SBET = 36.6	Vis	300 (Xe)	Na_2_S+Na_2_SO_3_	50 mg/100 mL	180	486.9	[92]
Bi_2_S_3_ (1.60 eV)/g-C_3_N_4_ (2.78 eV)	Solvothermal	Bi_2_S_3_ nanorods/g-C_3_N_4_ nanosheets S_BET_ = 58.1	Vis	300 (Xe)	Na_2_S + Na_2_SO_3_	30 mg/100 mL	180	101.8	[93]
g-C_3_N_4_ (2.85 eV)/CdSe-amine (1.86 eV)	Microwave solvothermal	g-C_3_N_4_ nanosheets/CdSe-amine flower like S_BET_ = 73.1	Vis	300 (Xe)	Na_2_S + Na_2_SO_3_	20 mg/50 mL	240	18.8	[94]
SnO_2_ (3.7 eV)/SnS_2_ (2.2 eV)	Solvothermal	SnO_2_ nanoparticles/SnS_2_ nanosheets S_BET_ = np	Vis	300 (Xe)	Pure water	np	180	5.5	[95]

* not provided.

## Data Availability

Data presented in this study are available by requesting from the corresponding author.
